# Physicochemical and biological impacts of light stress on adeno-associated virus serotype 6

**DOI:** 10.1016/j.omtm.2024.101362

**Published:** 2024-10-28

**Authors:** Rie Takino, Yuki Yamaguchi, Takahiro Maruno, Ekaputra Ramadhani, Misaki Furukawa, Tetsuo Torisu, Susumu Uchiyama

**Affiliations:** 1 Analytical and Quality Evaluation Research Laboratories, Daiichi Sankyo Co., Ltd, 1-2-58 Hiromachi, Shinagawa-ku, Tokyo 140-8710, Japan; 2 Department of Biotechnology, Graduate School of Engineering, Osaka University, 2-1 Yamadaoka, Suita, Osaka 565-0871, Japan; 3 Exploratory Research Center on Life and Living Systems (ExCELLS), National Institutes of Natural Sciences, 5-1 Higashiyama, Myodaiji-cho, Okazaki 444-8787, Japan

**Keywords:** adeno-associated virus, viral protein, encapsidated DNA, analytical ultracentrifugation, capillary gel electrophoresis, mass spectrometry, photodegradation, light stress

## Abstract

Recombinant adeno-associated virus (rAAV) is a small, nonenveloped virus vector that has attracted attention as a gene therapy platform. Several studies have revealed the stability characteristics of rAAV; however, its tolerance against light stress remains unclear. Here, the physicochemical and biological impacts of light stress on rAAV6 with EGFP as a gene of interest were investigated. The results revealed that light exposure led to an approximately 20% loss in particle number of rAAV with *s* value of full particles, whereas a more drastic decrease was observed in the genomic titer due to the degradation of encapsidated DNA, leading to an approximately 90% reduction in biological activity as evident from a flow cytometry-based assay. Structure analysis of the viral proteins VP1, VP2, and VP3 and the encapsidated DNA revealed that light exposure causes typical photodegradation of protein and DNA, but with AAV-specific phenomena. This included oxidation proceeding at histidine residues of VP3 on the inner surface of the capsid and the formation of cyclobutane pyrimidine dimer in DNA within the capsid. The findings highlight the necessity of the proper handling and storage to maintain the stability and efficacy of AAV-based gene therapies.

## Graphical abstract

**Figure:**
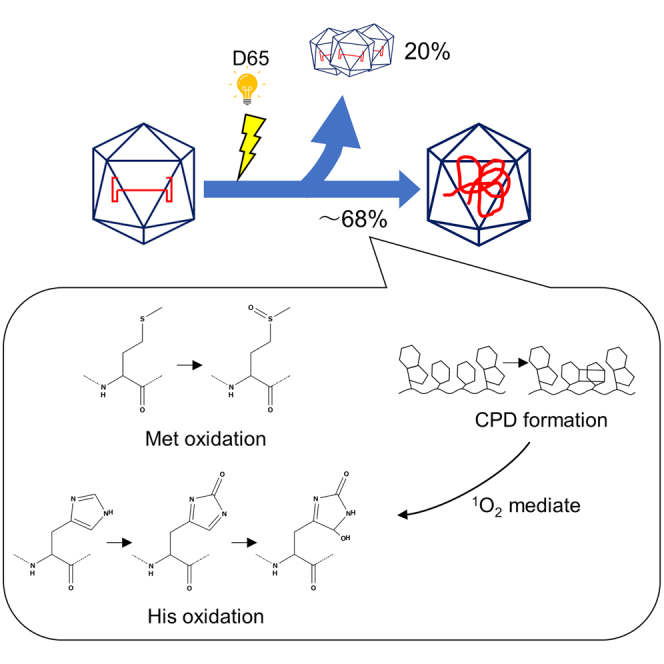

## Teaser

Uchiyama and colleagues revealed how light stress impacts the structure and function of AAV. Light exposure causes typical photodegradation of protein and DNA, but with AAV-specific phenomena. The full particle aggregation and degradation of the encapsidated DNA predominantly contributes to the loss of biological activity of AAV.

## Introduction

Adeno-associated virus (AAV) is a small, nonenveloped virus that belongs to the *Parvoviridae* family; it was identified in the 1960s.^1^^,^^2^^,^^3^ AAV has gained significant attention as a promising modality for *in vivo* gene therapy.^4^^,^^5^^,^^6^ Seven products have been launched, and there have been over 200 clinical trials worldwide using recombinant AAV (rAAV).^7^^,^^8^ rAAV comprises an icosahedral protein capsid and a recombinant DNA up to 4.7 kb in the case of a single strand. The capsid comprises three subunit proteins, VP1, VP2, and VP3, in a stoichiometric ratio of typically 5:5:50 for 60 subunits per capsid.^9^^,^^10^ Multiple serotypes have been identified and observed to exhibit different tissue tropisms,^11^^,^^12^ rendering AAV a more attractive modality for gene therapy.

Because these products are therapeutic drugs, their stability characteristics are essential information for the stable supply of a safe and efficient product. The stability characteristics of therapeutic proteins, such as monoclonal antibodies, have been intensively investigated against thermal, light, chemical, and physical stresses.^13^^,^^14^^,^^15^ In particular, information on thermal stability and photostability is considered more important and has been specified as mandatory for new drug applications in the International Council for Harmonisation of Technical Requirements for Pharmaceuticals for Human Use (ICH) Q1A to Q1E and Q5C guidelines.^16^ Degradation by light stress is known to occur in protein and DNA in nature and even in a controlled environment. Light conditions to which therapeutics may be exposed range from 1,000 lx in laboratories to 130,000 lx in direct sunlight.^17^^,^^18^ The most dominant spectral regions are the UVA (320–400 nm) and visible light (∼400–780 nm) regions. Various types of DNA damage, including base modifications, DNA strand breaks, cross-linking, and formation of DNA-protein adducts, have been reported.^19^^,^^20^ The common forms of DNA photodegradation include cyclobutane pyrimidine dimers (CPDs) and pyrimidine-pyrimidone (6-4) photoproducts. These are known to be induced by UVB and UVC, but several studies have indicated that CPDs can be directly induced even by weak powers of UV, UVA.^19^^,^^21^ Such lesions disrupt the normal base pairing and hinder DNA replication and transcription. From a pharmaceutical point of view, these degradations can compromise the safety and efficacy of drug products. Structural and functional changes have been reported that proteins undergo upon exposure to light. Various forms of photodegradation have been reported to lead to altered protein conformation, aggregation, and loss of therapeutic efficacy,^22^^,^^23^ with oxidation being the most common form of modification.^24^ Studies have shown that the susceptibility to photodegradation varies among proteins, with factors such as amino acid composition, structural stability, and solvent environment playing crucial roles. Under realistic conditions for therapeutics, above the 320-nm region, nonproteinogenic photosensitizers, which produce reactive oxygen species, are considered to play important roles as triggers of most protein modifications, since the amino acid side chains of tryptophan, tyrosine, phenylalanine, histidine, and cysteine solely absorb light in considerably shorter regions such as UV-C and UV-B.^25^^,^^26^ Previous studies using therapeutic antibodies indicate that methionine, histidine, and tryptophan residues are susceptible to oxidation under typical light stress.^24^^,^^27^

Despite its criticality, the stability characteristics of rAAV vectors have not been well elucidated. Recent studies have revealed that the thermal stability of AAV capsids varies among serotypes and have suggested that DNA release is dependent on its length.^28^^,^^29^^,^^30^^,^^31^ Furthermore, Howard and Harvey revealed that the transduction efficiency of rAAV1 decreased by 20% after storage at 4°C for over 7 weeks,^32^ and Xu et al. reported that DNA leakage increased with freeze–thaw stress.^33^ In addition, McKenna et al. reported the impact of pH on rAAV stability in relation to the phospholipase A2 (PLA2) domain of VP1u.^34^

In contrast to the existing literature on the thermal stability of rAAV, to the best of our knowledge, the impact of light stress on rAAV has only been reported from an efficacy perspective,^35^^,^^36^ not from the viewpoint of capsids or genome DNA degradation. Considering that rAAV has a more complex structure than therapeutic proteins do, comprehensive characterization of DNA and protein moieties is necessary to interrogate what portion and defect can affect the biological activity of rAAV. In this study, we evaluated the physicochemical and biological impact of light stress on rAAV6 containing genome-coding EGFP as a gene of interest (GOI). As expected, we observed degradations after light exposure in DNA and protein moieties. Our results indicate that the genome DNA in the capsids is susceptible to photodegradation and might mediate the degradation of the capsid protein. This study provides several novel insights from the detailed and comprehensive evaluation of light-stressed rAAV. These findings will contribute to the development of more stable products and/or formulations.

## Results

Light-stressed samples were prepared according to the light conditions prescribed by the ICH, and a comprehensive analysis was performed. The physicochemical analyses included particle evaluation by analytical ultracentrifugation (AUC) and droplet digital PCR (ddPCR), DNA moiety evaluation by capillary electrophoresis using laser-induced fluorescence (CE-LIF). In addition, protein moiety evaluation was performed via capillary gel electrophoresis (CGE) and liquid chromatography-mass spectrometry (LC-MS) to determine what degradation had occurred. The biological analysis included cell-based assays to evaluate the actual effect of light stress on rAAV function.

### Particle evaluation by AUC

**Figure 1:**
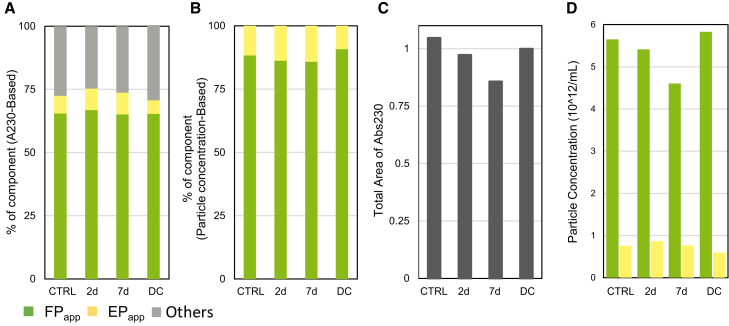
Changes in the particle profile of rAAV6 after light exposure measured via AUC Light-stressed rAAV6 samples were analyzed by AUC to obtain particle information. The entire component profile (A) and total peak area (C) were calculated based on the absorbance at 230 nm. Empty and full ratio (B) and particle concentration (D) were calculated from each peak area and extinction coefficient. CTRL, control sample; DC, dark control; EP_app_, apparent empty particle; FP_app_, apparent full particle.

Initially, we analyzed the light-stressed rAAV6 samples via AUC. Proteinaceous degradation can lead to fragmentation and/or aggregation, and there have been reports of encapsidated DNA being ejected under certain stress conditions.^33^ Here, AUC, a powerful tool that provides size distribution profiles of AAVs, including information on full (FP)/empty (EP)/partial particles and aggregates with their amounts/concentrations in absorbance units, was a good starting point.^37^ To address the common challenge of sample amount limitation for AAV analysis, we employed band sedimentation AUC techniques that require a smaller amount of AAVs compared with sedimentation velocity AUC.^38^ Throughout this study, AAVs with 98S and 69S were denoted as apparent FPs (FP_app_s) and apparent EPs (EP_app_s), respectively. Figure 1A shows that the initial population of FP_app_s, EP_app_s, and other components such as fragments or aggregates were 65.4%, 7.0%, and 27.6%, respectively. No significant trends were observed regarding the proportion of other components after light exposure. Only an approximate 2.5% decrease in the FP_app_ population in terms of the full/empty ratio (F/E, FP% in FP + EP) was observed after a 7-day exposure to light (Figure 1B). By contrast, a significant decrease in the total peak area at 230-nm absorbance was observed (Figure 1C). Interestingly, this decrease correlated well with a reduction in the FP_app_ concentration by 18.5% (Figure 1D). Even with the 18.5% decrease, the F/E ratio remained practically unchanged owing to the decrease in the total particle amount. Dynamic light scattering (DLS) analysis revealed an increase in large aggregates by light stress, which were not observed in the AUC analysis (Figures S1 and S2). No change was confirmed in the AUC and DLS analyses of the dark control (DC) sample.

### Genomic titer determined by ddPCR

**Figure 2:**
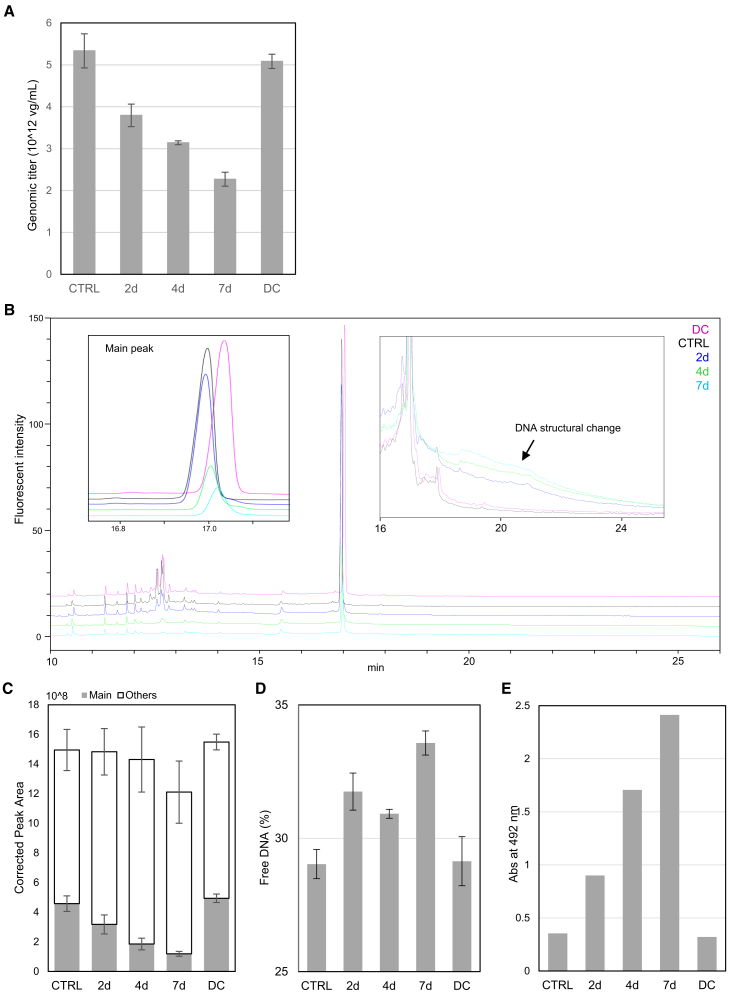
Photodegradation of encapsidated DNA of rAAV6 The genomic titers of light-stressed rAAV6 were measured by ddPCR (A). Stacked CE-LIF electropherograms of light-stressed rAAV6 are shown in (B), with an enlarged image of the main peak and the profile changes after the main peak. The quantitative results of CE-LIF are shown (C) for the main and other peak areas. Free DNA relative to the heat-ruptured control is shown in (D). The CPD ELISA results are shown in (E). For data shown in (C), the black, blue, green, cyan, and magenta lines represent the control (CTRL), 2-day light-stressed, 4-day light-stressed, and 7-day light-stressed and DC samples, respectively. Error bars represent the SDs of independent triplicate measurements for ddPCR and CE-LIF and triplicate measurements for free DNA.

We investigated the observed decrease in the AAV particles in the samples after a 7-day light exposure to elucidate what degradation had occurred. Several methods, such as ddPCR, qPCR, and ELISA, can be employed to estimate the “AAV concentration” based on different principles.^39^^,^^40^^,^^41^ Considering that AUC already provided information on particle concentration, we employed ddPCR to determine the genomic titer (Figure 2A). As expected, the genomic titer in the samples exhibited trends that were consistent with the AUC observation that the AAV concentration decreased after light exposure. However, the decrease in the genomic titer (57.4%) was more pronounced than that in the particle concentration estimated from AUC. No significant difference was observed in the genomic titer between the control and DC samples.

### Photostability of the encapsidated DNA

CE-LIF analysis on the encapsidated DNA was conducted to picture what type of degradation had occurred in the DNA. The CE profile of the encapsidated DNA was drastically altered by light exposure (Figure 2B). The corrected peak area (CPA) of the main peak decreased by 74.0% after a 7-day exposure to light, whereas the total CPA underwent a more moderate decrease (19.0%) in 7 days (Figure 2C). The second predominant peak migrated at 12–13 min and exhibited the same behavior as the main peak. No increase was observed in specific fragments. The electropherogram exhibited a gentle hill at the right side of the main peak in the light-stressed samples (Figure 2B), compensating for the main peak decrease. This 19.0% reduction in the total CPA was consistent with the AUC result, which showed an 18.6% reduction in FP_app_s. No significant difference was observed in the total CPA between the control and DC samples. In addition, samples without benzonase (endonuclease) treatment, which represent the total DNA in solution, including DNA outside capsids, exhibited a 69.5% decrease in the CPA of the main peak after 7 days of light exposure (Figure S3). The decrease in the CPA was close to 74.0%, which was obtained for the samples with benzonase treatment during the sample preparation for CE-LIF analysis. This was consistent with the results of a free DNA assay performed to confirm the ejection of the encapsidated DNA, with only approximately 4% increase in the free DNA after light exposure (Figure 2D). The ELISA results for CPD quantitation showed that CPD formation apparently was promoted by light exposure (Figure 2E).

### Photostability of protein moiety

**Figure 3:**
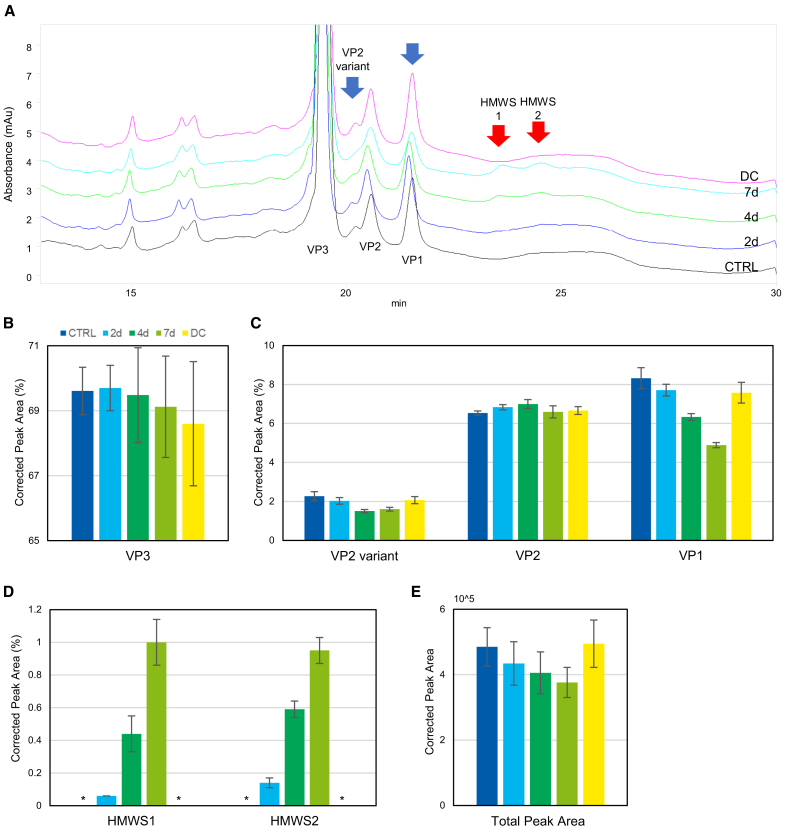
Changes in the VP profile of rAAV6 after light exposure measured by CGE Stacked CGE electropherograms of the light-stressed rAAV6 are shown in (A) with arrows for increased (red) and decreased (blue) peaks. Quantitative results of percentages for VP3 are shown in (B); VPx, VP2, and VP1 in (C); and HMWS in (D). Changes in the total peak area are shown in (E). For the data shown in (A), the black, blue, green, cyan, and magenta lines represent the control (CTRL), 2-day light-stressed, 4-day light-stressed, 7-day light-stressed, and DC samples, respectively. For data shown in (B)–(E), the blue and cyan, green, light green, and yellow bars represent the CTRL, 2-day light-stressed, 4-day light-stressed, 7-day light-stressed, and DC samples, respectively. Asterisks in (D) represent not detected. Error bars represent SDs of independent triplicate measurements.

Apart from DNA, another important AAV component is the protein capsid comprising VP1, VP2, and VP3. For a comprehensive analysis of the stability of AAV against light stress, we investigated the degradation of VPs. We observed several changes in the VP stoichiometric ratio during CGE analysis of the samples after light exposure (Figure 3A). Although VP1 and the peak between VP3 and VP2 (named VPx) decreased, several high-molecular-weight species (HMWS) increased after a 7-day light exposure (Figures 3B–3D). No other increase or decrease was observed. The total CPA decreased by 22.4% after a 7-day light exposure (Figure 3E), which was again consistent with the AUC and CE-LIF observations. No significant difference was observed in the total CPA between the control and DC samples.

### VPx and HMWS

**Figure 4:**
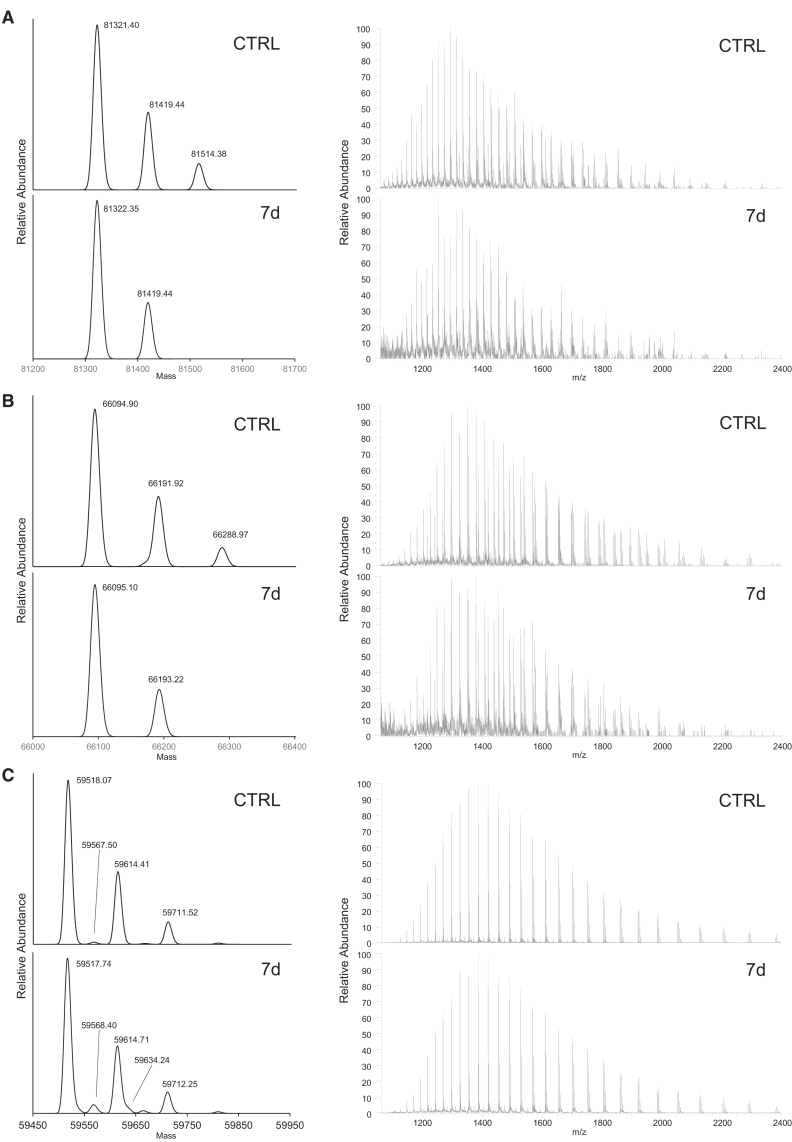
Intact MS analysis of rAAV6 after light exposure Deconvoluted and raw mass spectra are shown for VP1 (A), VP2 (B), and VP3 (C). Control (bottom) and 7-day light-stressed (top) samples are shown in each panel.

To elucidate the VPx and increased HMWS, LC-MS was performed. We observed VP1, VP2, and VP3, as well as other reported VP variants,^42^ with sufficient spectral quality and accuracy (Figure 4; Table 1). The +97-Da peak series was observed with each species. Based on the comparison of the MS data between the 0-day and 7-day light-stressed samples, a peak that decreased after a 7-day light exposure was identified with a deconvoluted mass of 61,290.75, between VP3 (59,518.07) and VP2 (66,094.93). Peptides are known to cleave at the DP or DG sequence under acidic conditions, and previous studies have reported the clipping VPs of AAV.^43^^,^^44^ Based on the amino acid sequence of AAV6, the sequence of VPx was identified as P185-L736, with a molecular mass of 61,291.18 Da, implying that the cleavage site in the VP2 region was the D/P sequence. The increased HMWS could not be detected during this intact MS analysis; however, SDS-PAGE analysis revealed bands of HMWS. Similarly, HMWS were confirmed in another serotype, AAV2 and AAV9 (Figure S4). The observed HMWS bands did not exhibit fluorescence signals when the gel was dyed with SYBR Gold, which stains nucleic acids, demonstrating that no covalent cross-linking existed between DNA and VP to form the detected HMWS.

**Table 1:** Deconvoluted mass list detected in LC-MS

	Predicted amino acid sequence	Actual amino acid sequence	Theoretical mass, Da	Control sample		7-day light-stressed sample	
				Measured mass, Da	Mass accuracy, ppm	Measured mass, Da	Mass accuracy, ppm
VP1	M1-L736	A2(Ac)-L736	81,322.18	81,321.40	−9.6	81,322.35	2.1
VP2	T138-L736	A139-L736	66,095.41	66,094.90	−7.7	66,095.10	−4.7
VP2 variant	P185-L736	P185-L736	61,291.18	61,290.20	−16.0	ND	ND
VP3	M203-L736	A204(Ac)-L736	59,519.10	59,518.07	−17.3	59,517.74	−22.8
VP3 variant	M211-L736	A212(Ac)-L736	58,890.40	58,889.19	−20.5	58,888.87	−26.0
VP3 fragment	M203-D626	A204(Ac)-D626	47,176.23	47,176.10	−2.8	47,175.66	−12.1
VP3 fragment	M203-D590	A204(Ac)-D590	43,221.69	43,221.16	−12.3	43,220.82	−20.1

ND, not detected.

### Post-translational modifications by light stress

**Figure 5:**
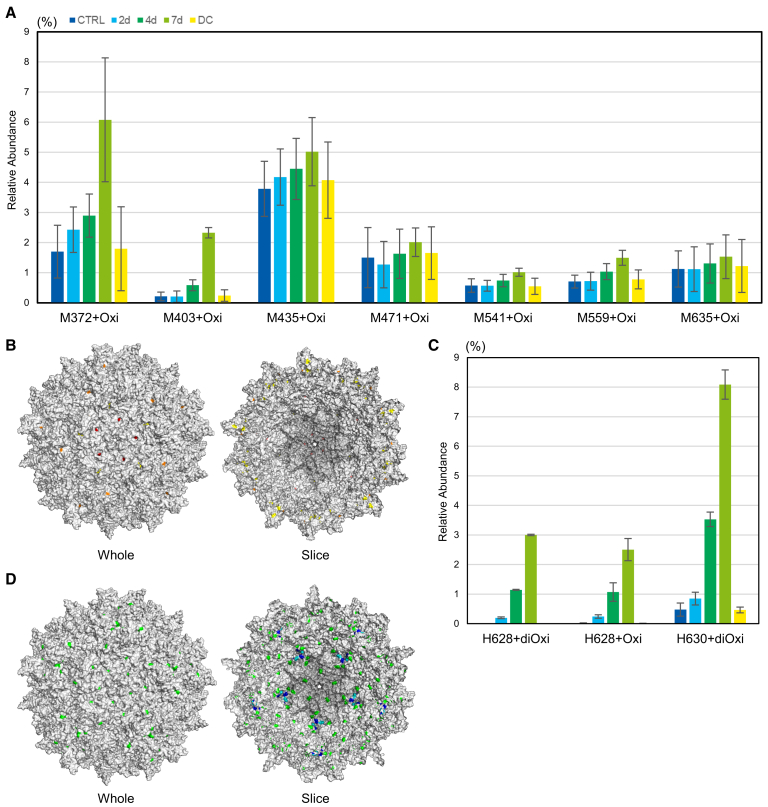
Changes in the oxidation of rAAV6 after light exposure Detected oxidation over 1.0% is summarized in (A) for methionine residues. The location of methionine residues in the capsid is shown in (B). Red, pink, and orange represent M372, M403, and M559, respectively, and yellow represents others. Detected oxidation over 1.0% is summarized in (C) for histidine residues. The location of histidine residues in the capsid is shown in (D). Blue and cyan represent H628 and H630, respectively, and green represents others. For data shown in (A) and (C), the blue and cyan, green, light green, and yellow bars represent the control (CTRL), 2-day light-stressed, 4-day light-stressed, 7-day light-stressed, and DC samples, respectively. Error bars represent the SDs of independent triplicate measurements.

Post-translational modifications (PTMs) induced by light stress were evaluated using pepsin-digested peptide mapping. Consistent with a previous study on nine therapeutic proteins,^24^ oxidation was observed at 7 out of 14 methionine residues, 3 of which were observed to increase in a light exposure-dependent manner (Figure 5A). The detected oxidation levels varied among the methionine residues. The oxidation levels at M372 and M403 were significantly elevated under light stress and remained stable in the DC sample. M435 and M559 exhibited a similar trend, albeit at a moderate level. The residues with increased oxidation levels were located on the inner and outer surfaces of the capsid, whereas the other methionine residues were buried in the structure (Figure 5B). Unexpectedly, of 16 histidine residues, only H628 and H630 were oxidized, and their oxidation levels increased after a 7-day light exposure (Figure 5C), although not only H628 and H630 but also the other histidine residues were located on the inner or outer surfaces of the capsid (Figure 5D). The oxidation of H628 and H630 included single oxidation (+16 Da) and double oxidation (+32 Da). The oxidation of tryptophan residues was detected; however, the oxidation level was relatively low compared with that of the methionine and histidine residues. In addition, the effect of light exposure was obscure (Figure S5).

Deamidation at N57 has been reported to impact AAV efficacy because this amino acid residue is positioned in a catalytic PLA2 domain of VP1.^45^^,^^46^^,^^47^ Studies have revealed that such deamidation proceeds by thermal stress. Here, deamidation at N57 increased in AAV6 in light-stressed samples; however, AAV6 in a DC sample was similarly deamidated at N57 during storage at 25°C, indicating that light exposure itself does not enhance the deamidation of AAV.

### Biological activity of light-stressed samples

**Figure 6:**
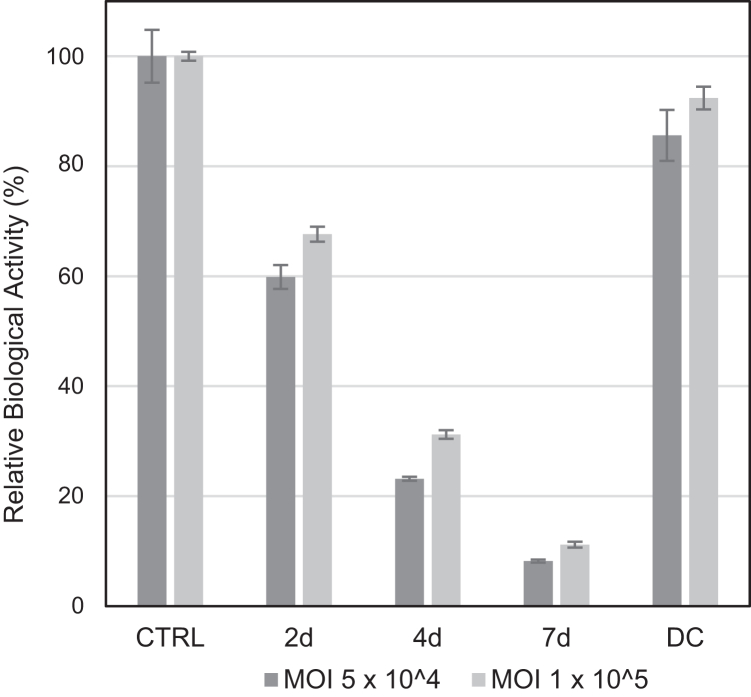
Changes in the biological activity of rAAV6 after light exposure The relative biological activities to the control (CTRL) sample are shown after light exposure. Error bars represent the SDs of triplicate measurements.

A cell-based assay was conducted to assess the impact of light stress on the biological activity of rAAV6. We used rAAV6 containing EGFP as a GOI; thus, the biological activities of light-stressed samples were measured by counting fluorescent cells after infection using a flow cytometer. The optimum MOI for this evaluation was determined based on a dose-response curve of the control (CTRL) sample (Figure S6). To achieve maximum sensitivity, the genomic titer slightly before the plateau was applied as the MOI for the evaluation of the light-stressed samples. Consistent with the previous studies, the biological activities of rAAV6 were dramatically compromised after a 7-day light exposure (Figure 6). Compared with the CTRL sample, the 7-day light-stressed sample exhibited only ∼10% biological activity.

## Discussion

### Particle loss

AUC revealed an approximately 20% reduction in the amount of FP_app_s, which was consistently and quantitatively confirmed by orthogonal methods, such as CGE and CE-LIF. The 20% reduction could be attributed to the formation of large aggregates. At the rotor speed used during the AUC, the large aggregates sedimented before the first scan in the AUC measurement, and it was therefore difficult to determine the exact size of the large aggregates. The DLS results showed an increase in aggregates in a light exposure time-dependent manner, although DLS is less quantitative, and a direct comparison of the quantity is challenging. Importantly, the stable EP_app_ concentrations observed in the AUC analysis indicated that the conversation of the FP_app_ to EP_app_ by releasing the genome was hardly occurred. Considering that the concentration of FP_app_s was significantly higher than that of the EP_app_s and that the F/E ratio after the light exposure was practically the same as that under the initial conditions, these results suggest that FP_app_s are more likely to be lost than EP_app_s. The F/E ratio is a common indicator of rAAV vector quality. However, the results of the F/E ratio estimation need to be carefully interpreted to avoid underestimating the impact when the amount of the total particles is changed, such as in a stability study.

A 57.4% decrease in the genomic titer was inconsistent with the decrease in particle concentration (up to 20%), even considering the differences in the methods employed. Thus, this result raised the possibility of additional reduction or degradation in the DNA, which would interfere with the PCR. As the AUC results indicated no increase in EP_app_s by light stress and the treatment of light-stressed samples with DNase prior to the ddPCR, it could be concluded that this degradation occurred in the encapsidated DNA without changes in the size, density, and shape of FP_app_s detectable with BS-AUC.

### DNA photodegradation

The CE-LIF analysis revealed a significant decrease in the CPA of the main peak, whereas the observed decrease in the total CPA was close to that of the FP_app_ reduction observed by AUC. This result suggests that the DNA derived from aggregates, which is the main cause of the approximately 20% FP_app_ loss, was not analyzed in CE-LIF and that the decrease in the CPA of the main peak in the light-stressed samples relative to the control sample contained the concentration difference caused by FP_app_ loss. The decrease in the CPA% of the main peak after 7 days of exposure was 67.8% relative to the control sample as the evaluation excluding the impact of particle loss, indicating that the DNA obtained from the particles excluding aggregates was highly damaged. Similarly, the result of the samples without benzonase treatment refutes the hypothesis that the release of encapsidated DNA is the main photodegradation of the AAV6 DNA moiety. The CPD ELISA assay revealed that the encapsidated DNA formed cross-links through light exposure. The D65 ramp does not contain wavelengths below 300 nm; thus, CPD formation was induced mainly by UVA (320–400 nm), as previously reported.^19^^,^^21^ Such covalent bonds and other possibly photoinduced degradants were likely to occur across the entire sequence length. Consequent conformational change in damaged DNA led to the alteration of its migration during the CE-LIF analysis. The sequences targeted by primers and a probe contain several sequential pyrimidine residues, as described in the materials and methods section. This can explain why the ddPCR using primers or probes targeting the cytomegalovirus (CMV) promoter could not properly amplify the targeted region, resulting in a significant decrease in the genome titer. The free DNA assay showed an increase of approximately 4% in free DNA; however, another serotype, rAAV9, exhibited minimal changes (Figure S7). It should be noted that the rAAV6 purchased for the present study had a relatively low genome purity owing to the presence of shorter DNA,^48^ and such shorter DNA fragments are known to exhibit higher mobility and are more easily released. Thus, it is unclear whether a common pattern of AAV DNA photodegradation exists among serotypes. However, short DNA fragments might have been released by light exposure, although at low levels, since the DC of rAAV6 did not exhibit significant changes in the free DNA levels.

### Protein photodegradation

CGE, intact MS, and peptide mapping were conducted to evaluate protein damage, and selective decreases in VP1 and VP2 fragments, the formation of HMWS, and increased oxidation levels of amino acid residues were observed. VPx was initially assumed to be a VP1 fragment containing a specific region of VP1, because its behavior was similar to that of VP1. However, a sequence containing the VP1 region was not identified. The VP2 fragment identified to have a close mass was formed by cleavage at the location with a sequence (D/P), which is prone to be cleaved in acidic condition.^43^^,^^44^ Considering that the sample was not subjected to acidic conditions during the sample preparation for CGE, VP2 fragments could be generated during a purification process in which affinity chromatography with acidic elution was applied. Interestingly, the changes in VP2 due to photodegradation were not confirmed by CGE. The +97 Da peak series for each VP was observed only when DMSO was present in the mobile phase and hence is likely a DMSO-related adduct. The formed HMWS was not evident from the chromatographic profile of intact MS. Possibly, the 20% aggregated FP_app_-derived VP was solubilized into the sample buffer during CGE and SDS-PAGE sample preparations owing to the presence of SDS. Although the observed HMWS were confirmed to be DNA-free protein components, the possibility of larger components (i.e., multiply cross-linked DNA and VPs) cannot be excluded owing to the limited separation range of the applied SDS-PAGE.

The oxidation of amino acid residues elicited expected and unexpected results. Methionine oxidation is a commonly reported reaction in protein photodegradation that is prone to occur. The photooxidation of methionine relies on photosensitizers, and our results in which the oxidized methionine residues were located on the surface of capsids are consistent with previous studies in that photosensitizers can access these residues. Compared with methionine, histidine oxidation does not occur frequently. Interestingly, H630 oxidized in the present study was reported to form a “DNA binding pocket” on the inner surface of a capsid.^49^ The observed oxidation was double oxidation, and singlet oxygen plays an important role in the double oxidation of histidine.^50^^,^^51^ Thus, the results suggest that encapsidated DNA underwent photodegradation, forming singlet oxygen, as previously reported,^52^ which led to the photooxidation of histidine in the vicinity of DNA including H628. Other studies using hydrogen/deuterium exchange MS revealed protection or conformational change around the 5-fold channel in the presence of DNA,^53^^,^^54^ evoking us histidine oxidation around the channel. Nevertheless, the oxidation of histidine residues around the 5-fold channel was not detected. It is unclear whether DNA interacts with capsid protein at a location distant from the channel, remotely inducing conformational changes around the channel or whether DNA interacts with capsid protein in the channel region but the distance between the histidine residues and DNA is too far to oxidize for the short lifetime of singlet oxygen of ∼4 μs.^55^

### Biological activity

Considering the analysis of the aforementioned physicochemical photodegradation characteristics, it is evident that the final biological activity decreases because of multiple factors, as observed in previous studies on other protein pharmaceuticals. However, the present qualitative and quantitative analysis revealed that the damage to the DNA in the capsid is the most significant degradation in the case of AAV. Here, 20% and 67.8% of the biologically effective particles were lost because of FP_app_ aggregation and the degradation of the DNA encapsidated in soluble FP_app_s, respectively, leaving only 12.2%, which was consistent with the observed biological activity of approximately 10%. PTMs may have contributed to the reduced biological activity; however, the impact is supposed to be minimal.

To estimate the impact of the light in a normal environment (e.g., 1,000 lx), the obtained results of the biological activity and DNA analysis were analyzed by regression to the first-order reaction equation (Figure S8; Table S1). This analysis showed that the photodegradation in these quality attributes was a pseudo-first-order reaction to the applied total light intensity, as confirmed in antibody protein photodegradation.^56^ With the obtained reaction rate constant (*k*), 20% reduction in biological activity related to the nonstressed sample (calculated as 61.04%) would occur after 88.6 h in a normal environment. A 24-h storage in such an environment would lead to an approximately 4% loss in biological activity.

Further research is required to determine the composition and cross-linking sites of HMWS. Additionally, the photodegradation conditions used here followed the ICH Option 1 conditions using D65 (natural light) as the light source. It is important to evaluate the impact of photodegradation under room lighting conditions, such as fluorescent lighting, considering the handling and storage of AAV. It has been reported that DNA photodegradation occurs with both UV light and visible light.^57^^,^^58^ However, the formation of pyrimidine dimers assumed in this study has been reported to occur with shorter-wavelength UV light. Thus, the photodegradation of AAV under room lighting conditions may lead to results different from those of this study.

### Conclusion

Here, the physicochemical and biological impacts of light stress from D65 lamps on the stability of rAAV6 particles were investigated. AUC, CGE for VPs, and CE-LIF for DNA consistently revealed that ∼20% of particle with *s* values of FPs was lost after a 7-day light exposure. Unexpectedly, the genomic titer analysis revealed a significant decrease, indicating the degradation of encapsidated DNA. CE-LIF analysis of the encapsidated DNA showed a drastic reduction in the main peak, which could have been caused by chemical linking in the DNA, such as CPD. Protein analysis revealed changes in the VP proportion and the presence of HMWS. The observed oxidation of methionine and histidine residues located at the surfaces of inner and outer capsids suggests that histidine residues in the vicinity of DNA are oxidized by the mutual effects of light stress. Cell-based assays confirmed a significant decrease in biological activity. Thus, the DNA moiety is the most fragile part of rAAV6 against light stress, and its degradation predominantly impacts the biological activity. These results are consistent with the existing knowledge on protein and DNA moieties and provide insights into the mutual effects between the protein and DNA of capsids. The study findings emphasize the importance of the proper handling and storage conditions of rAAV to maintain the stability and efficacy of AAV-based gene therapies.

## Materials and methods

### Materials

AAV6-CMV-GFP was purchased from SignaGen (Frederick, MD) at a concentration of 1.11 × 10^13^ vector genomes (vg)/mL of PBS with 0.005% PF-68. AAV2-CMV-GFP was purchased from VIROVEK (Hayward, CA) at a concentration of 2 × 10^13^ vg/mL of PBS with 100 mM sodium citrate and 0.001% PF-68. Both concentrations were determined by the manufacturers via qPCR.

### Methods

#### Preparation of light-stressed samples

A total of 300 μL rAAV6 was aliquoted to 2-mL cyclic olefin polymer vials (DAIKYO, Tochigi, Japan) and crimped using rubber stoppers (DAIKYO). The vials were placed in a thermo-chamber with a photometer (Nagano Science, Osaka, Japan or Espec, Osaka) controlled at 25°C, 60% relative humidity, and 4,000 lx from D65 lamps. DC vials were placed in the same chamber but within a paper box. All the samples subjected to analyses were passed through one freeze-thaw cycle after pulling from the stability chamber. The control sample was aliquoted from the same solution to the polypropylene tubes of rAAV6 and stored in a −80°C refrigerator.

#### ddPCR

The rAAV samples were incubated with DNase I (Takara, Shiga, Japan) at 37°C for 30 min to digest DNA outside of capsids. Following to EDTA (Nacalai, Kyoto, Japan) quenching at a final concentration of 50 mM, the capsids were denatured by heating at 95°C for 10 min. The DNA released from the capsids was diluted and subjected to ddPCR. The sequences of the primers and the probe (Takara) are as follows (5′-3′).

CMVp-forward primer: CATCAATGGGCGTGGATAGC

CMVp-reverse primer: GGAGTTGTTACGACATTTTGGAAA

CMVp-probe: [FAM] ATTTCCAAGTCTCCACCC [BHQ1]

Droplets were generated using an Auto-DG system (Bio-Rad Laboratories, Hercules, CA), following the manufacturer’s instructions. The droplets were placed in a QX200 ddPCR system (Bio-Rad) and subjected to 40 cycles of PCR with annealing at 60°C and 95°C denaturation, and each fluorescence was measured. All the other reagents and materials required for ddPCR were obtained from Bio-Rad.

#### Band sedimentation AUC

The AUC procedure precisely followed a recently reported reference procedure.^38^ Samples applied to sample sector fulfilled PBS/D_2_O+0.001% poloxamar 188 through a sample reservoir were analyzed at 20°C, 20,000 rpm with a radial increment of 10 μm every 150 s using Optima AUC (Beckman Coulter, Brea, CA) with UV detection. Each concentration of EP_app_s and FP_app_s was calculated from each peak area and extinction coefficient. Different extinction coefficients were applied to FP_app_ and EP_app_, as previously reported.^59^

#### CE-LIF for encapsidated DNA

Approximately 5 × 10^10^ vg of the rAAV samples, based on the genomic titer of the CTRL sample determined by ddPCR, was subjected to CE-LIF analysis. For the analysis of encapsidated DNA, the samples were treated with benzonase (Merck, Darmstadt, Germany) for 30 min at 37°C. After quenching with EDTA at a final concentration of 50 mM, the capsid protein was digested with Proteinase K for 1 h at 55°C, followed by heating for 20 min at 95°C. The encapsidated DNA was purified using the QIAquick PCR purification kit (Qiagen, Hilden, Germany), and the purified DNA was eluted with 30 μL of nuclease-free water (Thermo Fisher Scientific, Waltham, MA). For CE-LIF analysis, 20 μL of the eluate was mixed with 20 μL sample loading solution (Sciex, Framingham, MA) and incubated for 5 min at 70°C, followed by immediate placement on ice for 2 min. CE-LIF measurements were performed using a PA800Plus system (Sciex). The gel buffer, SYBR Green II RNA Gel Stain, and wash buffer were supplied in the RNA9000 Purity and Integrity kit (Sciex), and the measurements were mostly conducted following the manufacturer’s instructions. The injection setting was modified to 5.0 kV for 15 s to enhance sensitivity. Fluorescence signals from SYBR Green II bound to DNA were detected using an LIF detector at 488 nm excitation and 520 nm emission. The peak area was normalized using Equation 1:

(Equation 1) CPA=Ld×PA÷MT,

where *CPA* is the corrected peak area, *Ld* is the length to detector, PA is the peak area (unnormalized), and *MT* is the migration time.

#### Free DNA assay

The free DNA assay precisely followed a recently reported reference procedure.^60^ Samples were mixed with 1× SYBR Gold in a formulation buffer, and their fluorescence was measured at 495-nm excitation and 530-nm emission. Free DNA (%) was calculated relative to a control heat-ruptured sample at 85°C for 20 min.

#### CPD ELISA

CPD ELISA was conducted using the High Sensitivity CPD ELISA Kit (COSMO BIO, Tokyo, Japan) according to the manufacturer’s instructions, with minor modifications. The encapsidated DNA was extracted and purified from 2.7 × 10^10^ vg of the rAAV samples using the same CE-LIF procedure. Here, 30 ng of DNA, if 100% recovered, was added per well.

#### CGE for protein

The rAAV samples for CGE measurements were mostly prepared according to a reference procedure.^61^ A total of 600 ng of rAAV6 protein was denatured and buffer exchanged, following a protocol, and the final sample was diluted to 55 μL with water. CGE measurements were performed using a PA800Plus system (Sciex). The prepared samples were injected with water plug sample stacking. Detection was performed at 214 nm using a photodiode array detector. The peak area was normalized using Equation 1.

#### LC-MS for peptide mapping

The rAAV samples for peptide mapping were prepared following a reference procedure, with minor modifications.^62^ Approximately 3 μg rAAV protein and the Pepsin SMART Digest buffer (pH 2) provided with SMART Digest Pepsin Kits (Thermo Fisher Scientific) were mixed at a ratio of 2:1 (v/v). Tris (2-carboxyethyl) phosphine (FUJIFILM Wako Chemicals, Osaka, Japan) was added to the digestion mixture at a final concentration of 12 mM. A total of 10 μL of the Magnetic SMART digest resin containing 9 μg heat-stable immobilized pepsin was added to the sample, and peptic digestion proceeded at 70°C for 45 min at 1,400 rpm. After the digestion, the supernatant was collected using a magnetic stand. All the samples were acidified using formic acid (final concentration 0.1%), and 50 μL of the digest mixture was loaded onto the column for chromatographic analysis. The peptide mixture was separated by reversed phase-high-performance LC (HPLC) using an ACQUITY UPLC (ultra-performance LC) BEH C18 Column (130 A, 1.7 μm, 1 × 100 mm, Waters, Milford, MA) and detected by Orbitrap-MS (Exploris 240, Thermo Fisher Scientific). Data analysis was conducted using BioPharma Finder (version 4.1, Thermo Fisher Scientific). Peptide identification was performed against the AAV6 VP1 viral capsid protein sequences with MS and MS/MS tolerances of 5 ppm. Equation 2 was used to calculate the abundance of PTMs using ions above the intensity threshold for peptide (>17%) relative to the most abundant signal and for charge states (>33%) relative to the most abundant charge state signal.

(Equation 2) %Abundance=(SumofMSareasforallmodifiedcomponents)/(SumofMSareasforallselectedcomponents)×100

#### LC-UV-MS for intact VPs

The rAAV samples were injected into Nexera X2 HPLC (Shimadzu, Kyoto, Japan) coupled with Orbitrap-MS. The injected VPs were separated using an ACQUITY UPLC PrST C4 Column, (300 A, 1.7 μm, 2.1 × 100 mm, Waters) at a flow rate of 0.2 mL/min and 80°C. Mobile phases A and B were 0.1% difluoroacetic acid (Waters), 5% DMSO in water, and acetonitrile (FUJIFILM Wako Chemicals), respectively. Intact VPs were eluted from 30% B to 34% B gradient in 15 min. UV absorbance at 280 nm was monitored. Deconvolution analysis was performed using BioPharma Finder.

#### SDS-PAGE

The rAAV samples were diluted and mixed with 4× lithium dodecyl sulfate sample buffer (Thermo Fisher Scientific) and 10× reducing agent (Thermo Fisher Scientific) to a final concentration of 20 ng/μL protein based on a measurement using a bicinchoninic acid kit (Thermo Fisher Scientific). The mixtures were incubated for 10 min at 70°C. The samples and ladder solution (BenchMark Protein Ladder, Thermo Fisher Scientific) were loaded to a 4%–12% Bis-Tris gel (Thermo Fisher Scientific) and run in 1× 3-(*N*-morpholino)propanesulfonic acid buffer (Thermo Fisher Scientific) for 5 min at 200 V and then for 50 min at 150 V. Imperial stain (Thermo Fisher Scientific) was used to visualize the bands according to the manufacturer’s instructions.

#### *In vivo* transduction assay

HeLa RC32 cells (American Type Culture Collection [ATCC], Manassas, VA) were seeded at 1 × 10^5^ cells/500 μL in 12-well plates and incubated for 1 day. The incubated cells were treated with rAAV6 samples at an MOI of 5 × 10^4^ or 1 × 10^5^ in 500 μL of DMEM (Sigma-Aldrich, St. Louis, MO) supplemented with 10% fetal bovine serum (ATCC) for infection, and 500 μL DMEM was added to the wells after 4 h. After incubation for 2 days, cells were harvested and their GFP expression was measured with CytoFLEX (Beckman Coulter).

## Data and code availability

The data shown in this article are available on request.

## Acknowledgments

This study was supported by a grant-in-aid from the Research and Development of Core Technologies for Gene and Cell Therapy supported by the Japan Agency for Medical Research and Development (10.13039/100009619AMED) (grant number JP18ae0201002). The authors would like to thank Nobuyuki Suzuki, Ph.D., Tetsuya Araki, Ph.D., and Masayoshi Shibata (Daiichi Sankyo Company) for their useful advice and great discussions.

## Author contributions

R.T.: conceptualization, investigation, writing – original draft, and visualization. T.M., E.R., and M.F.: investigation. T.T. and Y.Y.: writing – review & editing and supervision. S.U.: conceptualization, funding acquisition, writing – review & editing, supervision, and project administration.

## Declaration of interests

R.T. is an employee of Daiichi Sankyo Company.

## Declaration of generative AI and AI-assisted technologies in the writing process

During the preparation of this work the authors used DeepL Translate and Chat GPT to improve readability and language. After using the tools, the authors reviewed and edited the content as needed, and they take full responsibility for the content of the publication.

## Supplemental information

Document S1. Figures S1–S8 and Table S1

Document S2. Article plus supplemental information
