# Supplementary material for: Physicochemical and biological impacts of light stress on adeno-associated virus serotype 6
Source: Mol Ther Methods Clin Dev. 2024 Oct 28;32(4):101362. doi: 10.1016/j.omtm.2024.101362 (PMC11609365; doi:10.1016/j.omtm.2024.101362)
Supplement: Document S1. Figures S1–S8 and Table S1 [file mmc1.pdf]

OMTM, Volume *32*

Supplemental information

Physicochemical and biological impacts of light stress on adeno-associated virus serotype 6

Rie Takino, Yuki Yamaguchi, Takahiro Maruno, Ekaputra Ramadhani, Misaki Furukawa, Tetsuo Torisu, and Susumu Uchiyama

Supplemental Information

Tables

Table S1. First reaction constant of each degradation under light stress

	First reaction constant k (10^{-6} lx^{-1})	Changes after 24-h storage in a normal environment (1000 lx/h)
Biological activity (%)	2.82 (±0.40)	−5.1
DNA main peak area (%)	1.92 (±0.22)	−1.4
CPD ELISA	1.44 (±1.24)	+0.13

Figures

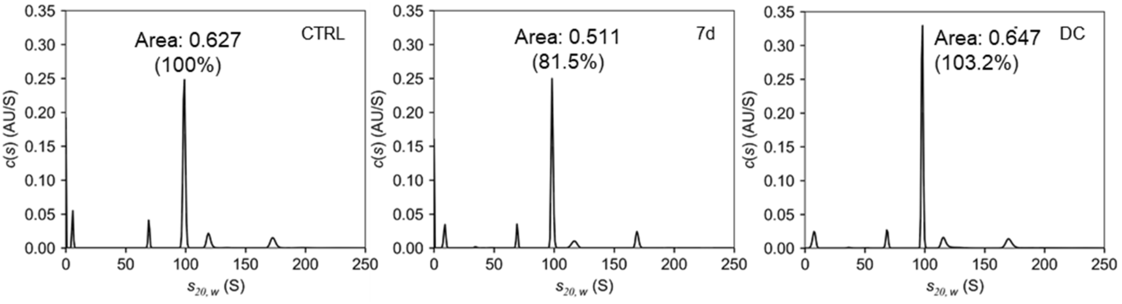

Figure S1. Sedimentation coefficient distribution profiles

No apparent change in dispersion state was observed between the control samples and light-stressed samples. Peak area of FP_{app} and relative percentage to the control sample in each annotation. CTRL: control sample, DC: dark control sample.

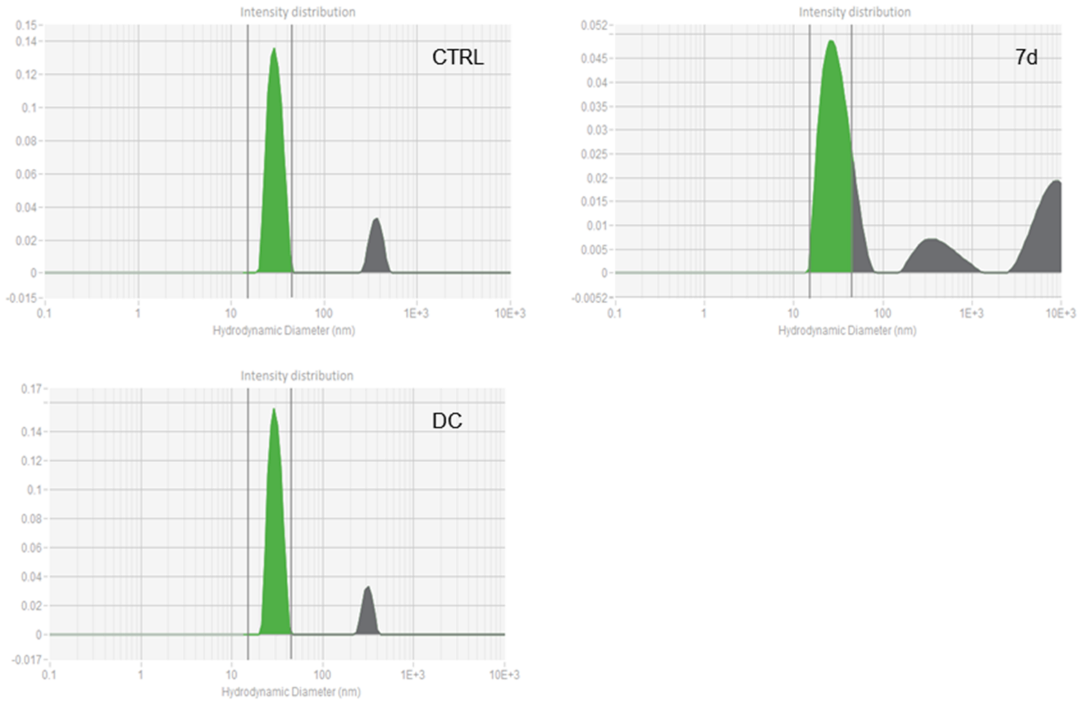

Figure S2. Large aggregation induced by light exposure

The DLS results showed larger aggregates over 1000 nm and a broad peak for the AAV monomer around 25–30 nm in a 7-day light-stressed sample compared with the control (CTRL) and the dark control (DC) samples.

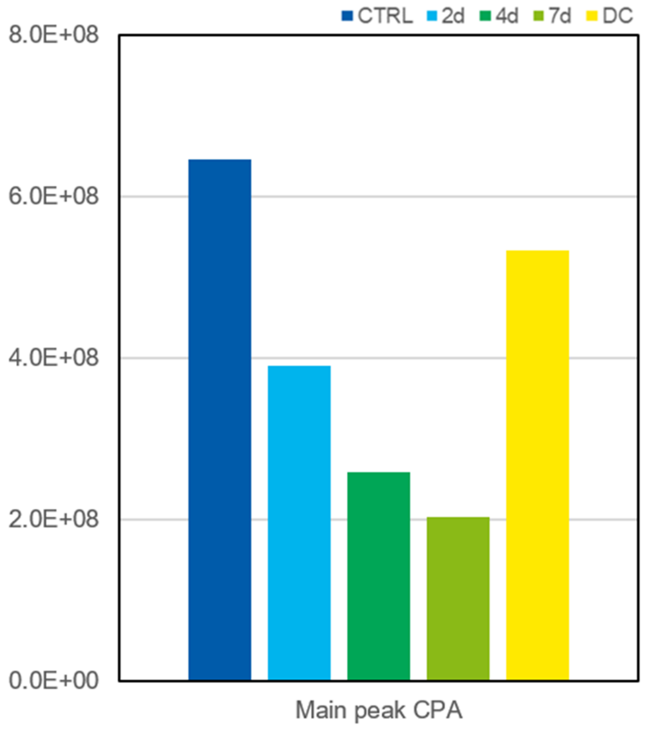

Figure S3. Reduction in DNA main peak without benzonase treatment

The samples without benzonase treatment were analyzed with CE-LIF, showing a 69.5% decrease in the CPA of the main peak after 7 days of light exposure. The blue, cyan, green, light green, and yellow bars represent the control (CTRL), 2-day light-stressed, 4-day light-stressed, 7-day light-stressed, and dark control (DC) samples, respectively.

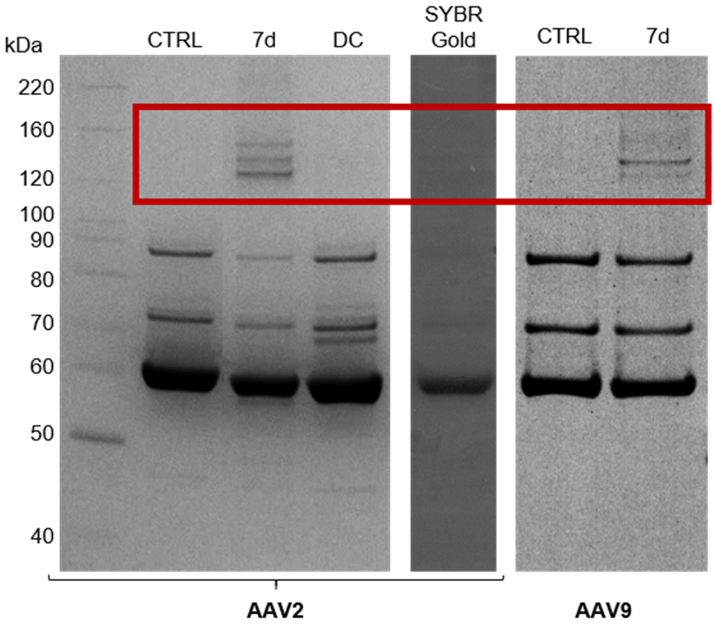

Figure S4. HMWS of VPs in light-stressed samples

SDS-PAGE analyses showed that AAV2 and AAV9 formed HMWS after a 7-day light exposure. No fluorescent band was detected by SYBR Gold staining, demonstrating no DNA covalent interaction in the HMWS formation.

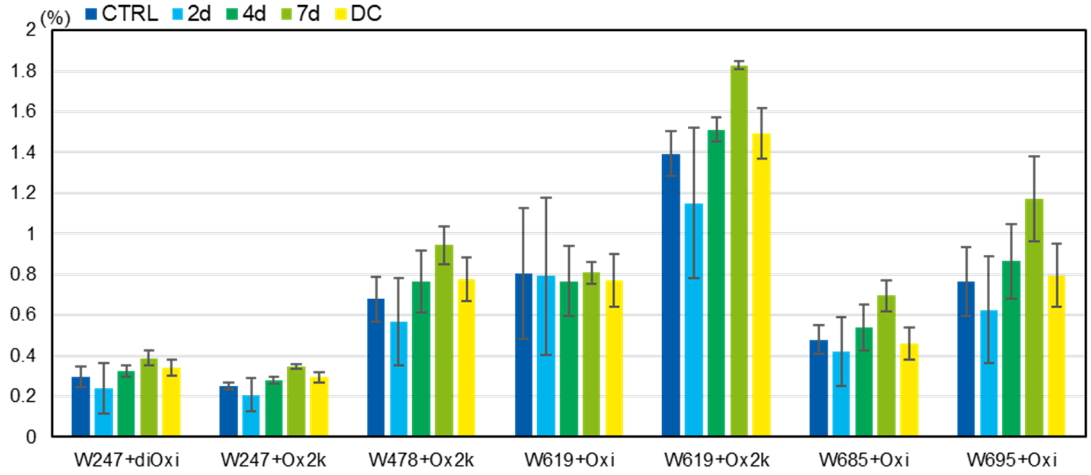

Figure S5. Tryptophan oxidation in light-stressed samples

The detected oxidation was summarized for the tryptophan residues. Only W619 and W695 showed over 1.0% oxidation in the 7-day light-stressed sample. The blue, cyan, green, light green, and yellow bars represent the control (CTRL), 2-day light-stressed, 4-day light-stressed, 7-day light-stressed, and dark control (DC) samples, respectively.

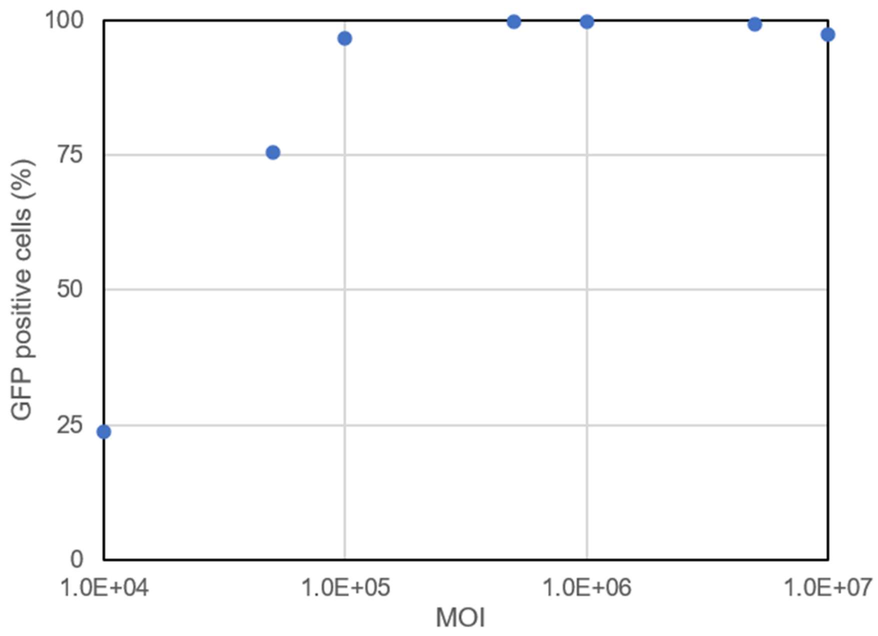

Figure S6. Dose-response curve of control sample

The GFP-positive cells (%) in the total cells were plotted against the MOIs to obtain a dose-response curve of the non-stressed control sample. The MOIs for the evaluation of the light-stressed samples were determined from the curve.

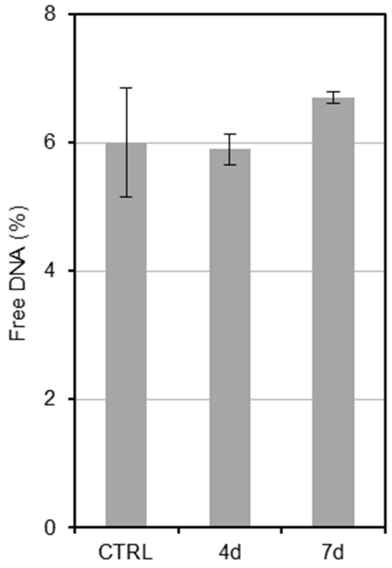

Figure S7. DNA leakage of rAAV9 by light stress

The free DNA (%) of rAAV9 was stable after a 7-day light exposure.

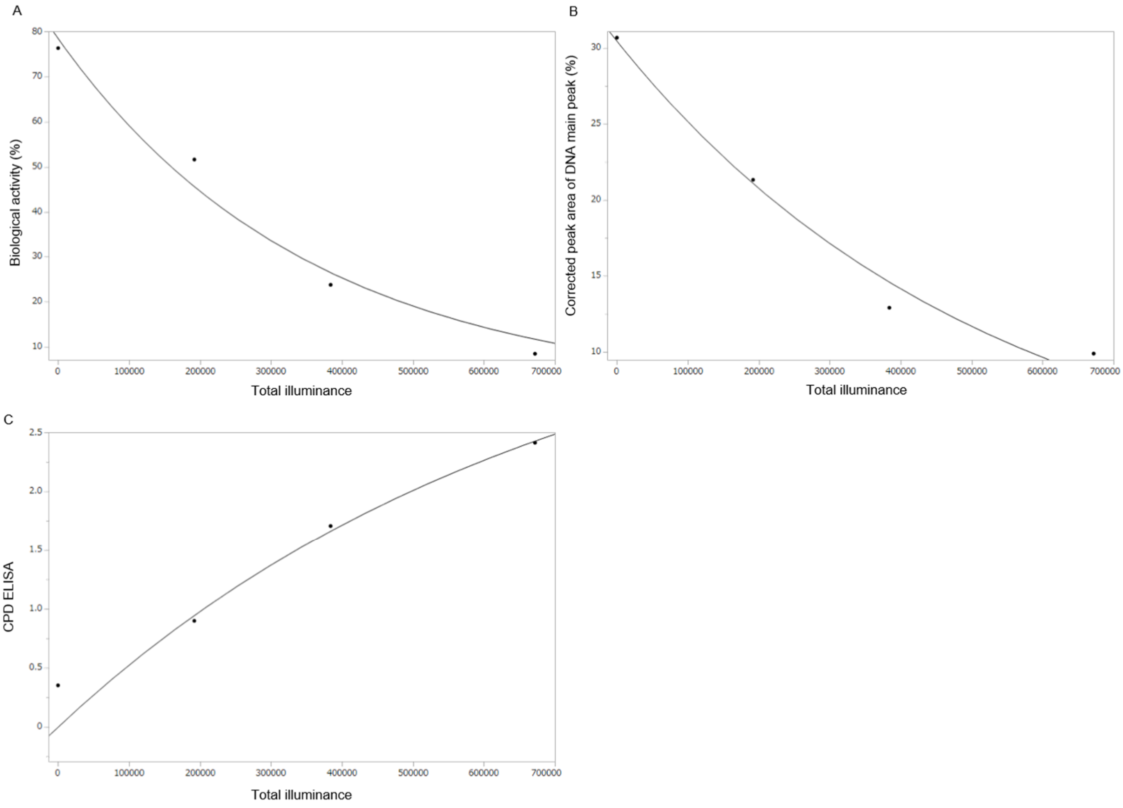

Figure S8. Changes caused by light stress in (A) the biological activity (%), (B) DNA main peak area (%), and (C) CPD ELISA

Each result was analyzed by regression to the first-order reaction equation. For the biological activity (%) and DNA main peak (%), $c = A_0 \cdot e^{-kt}$. For CPD ELISA, $c = A_0 \cdot (1 - e^{-kt})$. Here, c is each level after degradation, A_0 is the initial level of each item, *k* is a first-order reaction constant (lx^{-1}), and t is the total illuminance (lx).
